# Variable impact of the hemagglutinin polybasic cleavage site on virulence and pathogenesis of avian influenza H7N7 virus in chickens, turkeys and ducks

**DOI:** 10.1038/s41598-019-47938-3

**Published:** 2019-08-09

**Authors:** David Scheibner, Reiner Ulrich, Olanrewaju I. Fatola, Annika Graaf, Marcel Gischke, Ahmed H. Salaheldin, Timm C. Harder, Jutta Veits, Thomas C. Mettenleiter, Elsayed M. Abdelwhab

**Affiliations:** 1 Institute of Molecular Virology and Cell Biology, Friedrich-Loeffler-Institut, Federal Research Institute for Animal Health, Südufer 10, 17493 Greifswald-Insel Riems, Greifswald, Germany; 2 Department of Experimental Animal Facilities and Biorisk Management, Friedrich-Loeffler-Institut, Federal Research Institute for Animal Health, Südufer 10, 17493 Greifswald-Insel Riems, Greifswald, Germany; 3 Institute of Diagnostic Virology, Friedrich-Loeffler-Institut, Federal Research Institute for Animal Health, Südufer 10, 17493 Greifswald-Insel Riems, Greifswald, Germany

**Keywords:** Molecular evolution, Influenza virus

## Abstract

Avian influenza viruses (AIV) are classified into 16 hemagglutinin (HA; H1-H16) and 9 neuraminidase (NA; N1-N9) subtypes. All AIV are low pathogenic (LP) in birds, but subtypes H5 and H7 AIV can evolve into highly pathogenic (HP) forms. In the last two decades evolution of HPAIV H7 from LPAIV has been frequently reported. However, little is known about the pathogenesis and evolution of HP H7 from LP ancestors particularly, in non-chicken hosts. In 2015, both LP and HP H7N7 AIV were isolated from chickens in two neighbouring farms in Germany. Here, the virulence of these isogenic H7N7 LP, HP and LP virus carrying a polybasic HA cleavage site (HACS) from HP (designated LP-Poly) was studied in chickens, turkeys and different duck breeds. The LP precursor was avirulent in all birds. In contrast, all inoculated and contact chickens and turkeys died after infection with HP. HP infected Pekin and Mallard ducks remained clinically healthy, while Muscovy ducks exhibited moderate depression and excreted viruses at significantly higher amounts. The polybasic HACS increased virulence in a species-specific manner with intravenous pathogenicity indices of 3.0, 1.9 and 0.2 in chickens, turkeys and Muscovy ducks, respectively. Infection of endothelial cells was only observed in chickens. In summary, Pekin and Mallard were more resistant to HPAIV H7N7 than chickens, turkeys and Muscovy ducks. The polybasic HACS was the main determinant for virulence and endotheliotropism of HPAIV H7N7 in chickens, whereas other viral and/or host factors play an essential role in virulence and pathogenesis in turkeys and ducks.

## Introduction

Avian influenza A viruses (AIV) are members of the family *Orthomyxoviridae*. They are differentiated according to the antigenicity of the hemagglutinin (HA) and neuraminidase (NA) proteins into 16 HA (H1 to H16) and 9 NA (N1 to N9) subtypes^1^. HA is a surface glycoprotein which mediates virus attachment and fusion with the host cell endosomal membrane. It plays essential roles in virulence, immunogenicity and interspecies transmission (e.g. from birds to mammals)^2^. The HA is synthesized in a fusion-inactive form (HA0), which is activated through cleavage by host proteases into HA1 and HA2 subunits. The HA monobasic cleavage site (CS) of low pathogenic (LP) AIV is recognized by trypsin-like proteases in the respiratory and digestive tracts. LPAIV cause mild, if any, clinical signs, while highly pathogenic (HP) AIV carry a multibasic CS, which is cleaved by ubiquitous furin-like proteases resulting in systemic infections and high mortality^3–6^. In nature, only H5 and H7 subtypes can evolve from LP precursors to HPAIV by acquisition of mutations particularly in CS of the HA protein^4^. This transition mostly occurs in terrestrial poultry but can spill to wild birds^7^. Evolution of HPAIV from LPAIV is usually accompanied by mutations in other gene segments in addition to HA. These mutations are unique to each HPAIV and can modulate virulence and bird-to-bird transmission^8–12^. Therefore, it is important to study the virulence determinants of each HPAIV separately.

Recently, the prevalence of H7 viruses and the evolution of HPAIV H7 from low pathogenic precursors increased remarkably^13–17^. Turkeys are more susceptible than chickens to AIV, enabling adaptation of wild bird-origin viruses to other domestic birds and play an important role in interspecies transmission (e.g. from birds to pigs and humans and *vice versa*)^18–20^. Nevertheless, data on the virulence determinants of HPAIV in turkeys are scarce. Likewise, few studies have been conducted in domestic ducks to evaluate the pathogenicity of HPAIV H7Nx. Several HPAIV H7N3 from Chile, Canada and Mexico, H7N4 from Australia and H7N7 from the Netherlands did not cause any mortality in two-week-old Pekin ducks^21^. Similarly, Pekin and Mallard ducklings were clinically resistant to the Italian HPAIV H7N1^21,22^. Commercial young or adult Pekin ducks did not succumb to mortality with different Dutch and Australian HPAIV H7N7^23–26^. Virulence of HPAIV H7 in Muscovy ducks has not been studied before, although under natural conditions neurological disorders and high mortality during the 1999–2000 outbreaks in Italy were linked to infection by HPAIV H7N1^27^. Muscovy ducks were more sensitive to HPAIV H5N1 than Pekin ducks^28–30^, probably due to differences in immune responses^29,30^ which may vary according to the virus strain, age of ducks and route of virus inoculation^28,31,32^. Importantly, virulence determinants of HPAIV H5Nx in chickens differ from those in ducks^33–36^.

In 2015, an HPAIV H7N7 was isolated from a commercial chicken layer flock in Germany. The virus evolved from LP H7N7, which was simultaneously isolated from the same farm. The HA of the low pathogenic virus carried a monobasic HACS (PEIPKGR/G), while the HP specified a polybasic CS (PEIPKRKRR/G)^37^. Here, the impact of the polybasic HACS on virus replication, virulence, bird-to-bird transmission and tropism was investigated in chickens, turkeys and different duck breeds.

## Results

### Three recombinant H7N7 viruses were successfully generated using reverse genetics

All gene segments of LPAIV A/chicken/Germany/AR915/2015(H7N7) (designated hereafter LP) and HPAIV A/chicken/Germany/AR1385/2015(H7N7) (designated hereafter HP) were successfully amplified and cloned. Moreover, to generate an LP with a polybasic CS (designated hereafter LP-Poly), the HA segment of LP was used to change the monobasic (PEIPKGR/G), to the polybasic PEIPKRKRR/G, exactly resembling the HP H7N7 CS. LP, HP and LP-Poly were successfully rescued and showed comparable titres from 10^6.7^ to 10^7.0^ plaque forming unit per ml (PFU/ml) in the allantoic fluids after propagation in specific pathogen free (SPF) embryonated chicken eggs (ECE).

### The impact of a polybasic HACS on virus replication, spread and HA cleavability in cell culture

**Figure 1:**
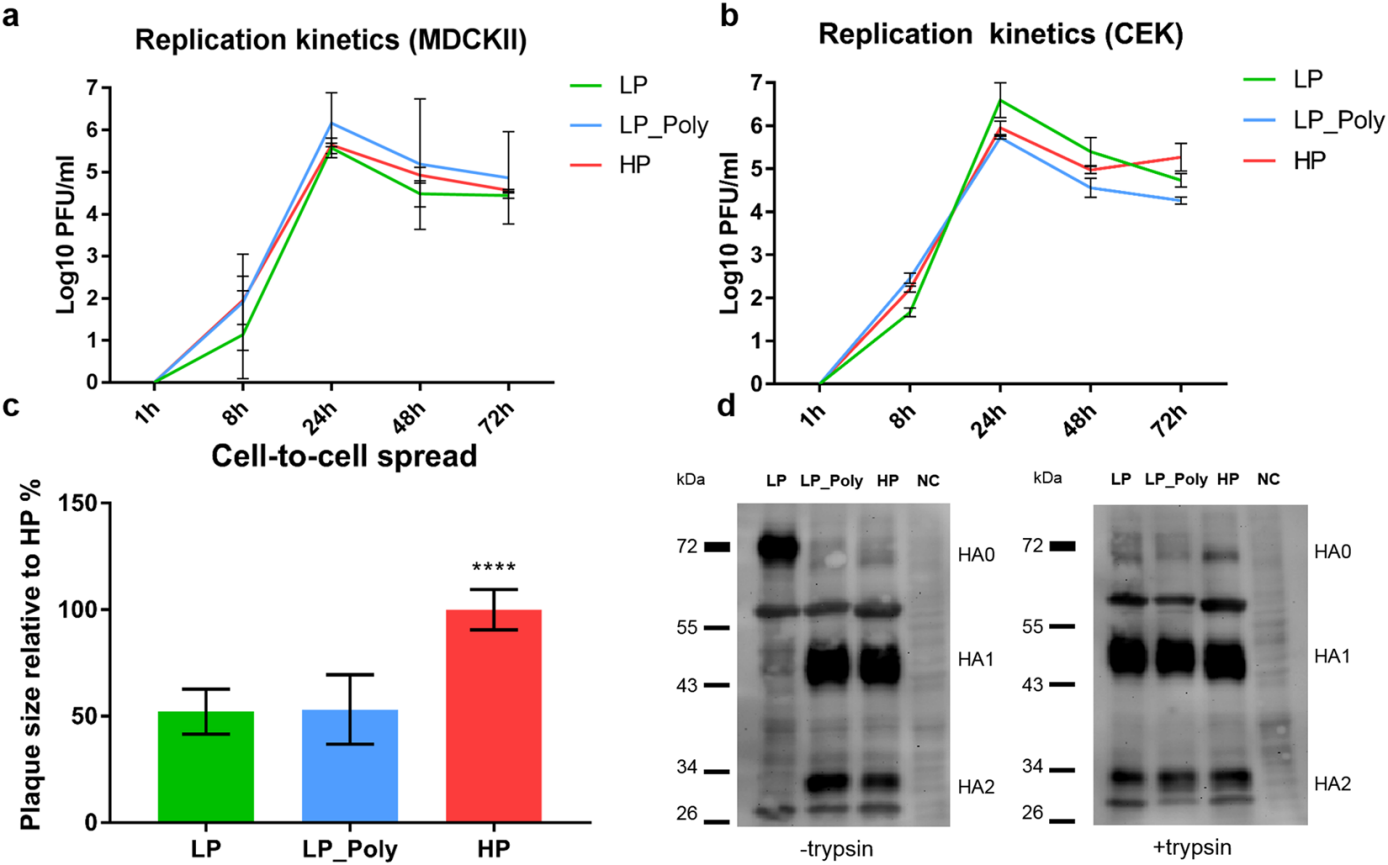
*In vitro* characterisation of recombinant H7N7 viruses in this study. The replication efficiency in MDCKII (**a**) and CEK (**b**) cells at an MOI of 0.001 for 1, 8, 24, 48 and 72 hours post infection is illustrated. Results are shown as the mean and standard deviations of all values of triplicates of two independent runs. Cell-to-cell spread was determined by measuring the diameter of plaques induced by the indicated viruses in MDCKII cells. The size of plaques induced by HP was set as 100%. The results are expressed as mean and standard deviation. (**c**) The cleavability of HA0 into HA1 and HA2 subunits was studied using Western Blot after the infection of MDCKII cells at an MOI of 1 PFU per cell of indicated viruses in the presence (+) or absence (−) of trypsin. The viral proteins were detected by polyclonal chicken serum against H7N1 at a ratio 1:500 after separation in a 10% polyacrylamide gel. NC refers to the mock control (non-infected cells). (**d**) The original Western Blot figure is available as a Supplementary Fig. S1.

All three viruses replicated in avian and mammalian cell lines. No significant differences were observed in chicken embryo kidney (CEK) cells (Fig. 1 panel b). In Madin-Darby canine kidney cells type II (MDCKII), LP-Poly replicated to higher titres than the LP and HP viruses (Fig. 1 panel b), which, however, was not statistically significant (P > 0.05). The HP virus induced significantly (P < 0.0001) larger plaques in MDCKII than LP and LP-Poly (Fig. 1 panel c). The HA of LP was only efficiently activated in the presence of trypsin, while insertion of a polybasic cleavage site allowed cleavage of the LP HA in the absence of trypsin (Fig. 1 panel d).

### Insertion of the polybasic CS increased virulence and tropism of LP H7N7 comparable to HP H7N7 after oculonasal (ON) or intravenous (IV) infection of chickens

**Figure 2:**
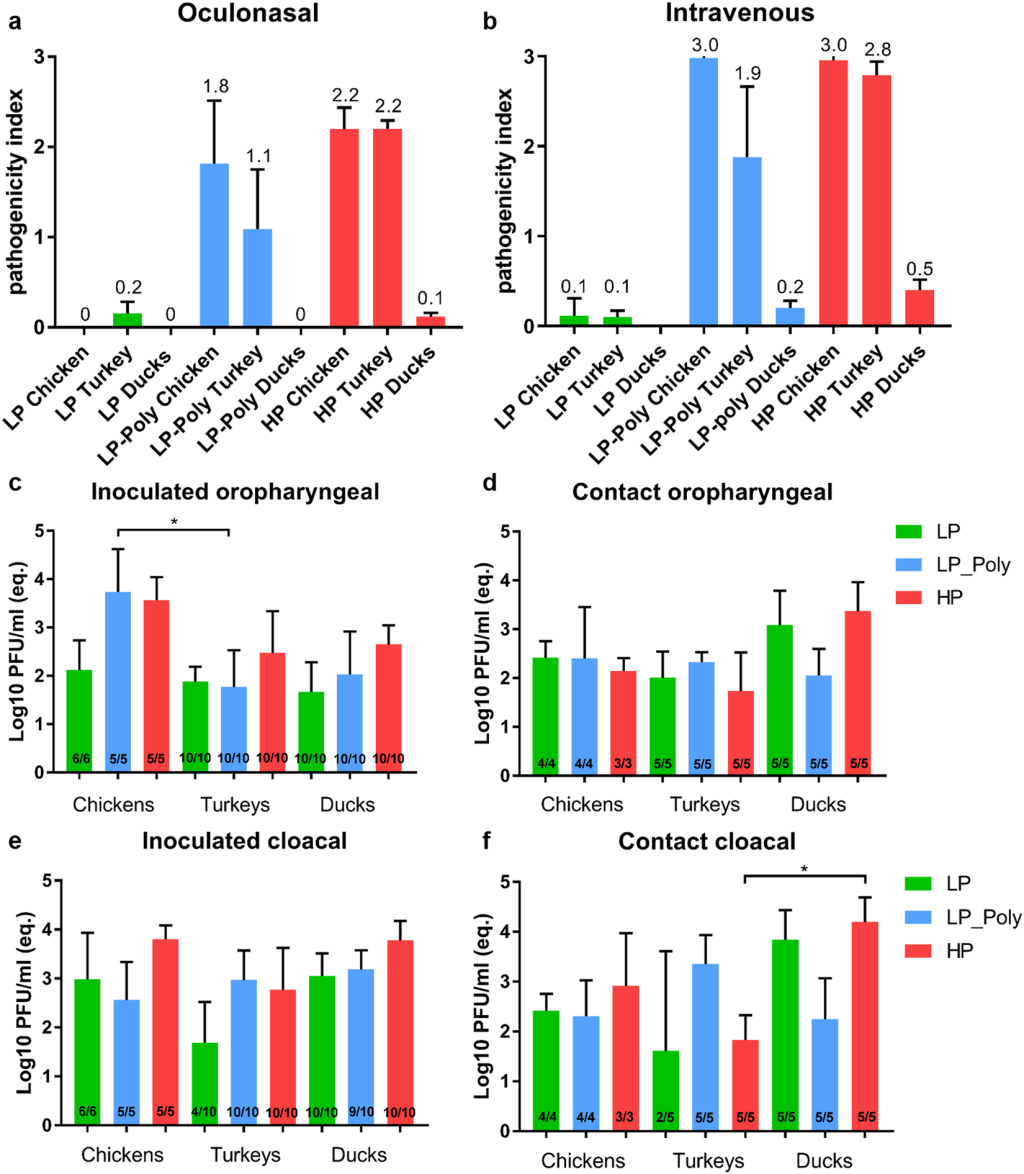
Clinical scoring and virus excretion in experimentally infected chickens, turkeys and Muscovy ducks. Clinical scoring after oculonasal (**a**) or intravenous (**b**) infection was calculated by dividing the sum of the arithmetic mean values of daily scores by 10 (the number of observation days). The PI and IVPI values for each virus ranged from 0 (avirulent) to 3 (highly virulent) and the results are expressed as mean and standard deviation. Virus excretion at 4 days post inoculation in oropharyngeal (**c**,**d**) and cloacal (**e**,**f**) swabs of inoculated (c and e) and contact (d and f) birds was determined by RT-qPCR against standard curves using ten-fold dilutions of HPAIV H7N7 and the mean and standard deviations were expressed as equivalent plaque forming unit pro ml (eq. PFU/mL). Asterisks indicate significant differences at p ≤ 0.05 (*) or 0.01 (**) or 0.0001 (****).

After oculonasal (ON) inoculation with LP, chickens showed mild clinical signs (i.e. ruffled feathers and diarrhoea) for a few days and recovered quickly. All contact birds in this group survived without showing clinical signs. Conversely, 5/6 and 6/6 inoculated chickens, and 3/4 and 4/4 contact chickens died after infection with LP-Poly and HP viruses with pathogenicity indices (PI) of 1.8 and 2.2, and a mean death time (MDT) of 5 and 4.3 days, respectively (Table 1, Fig. 2 panel a). Birds showed clinical signs typical of HPAIV infection, e.g. cyanosis of comb and wattle, haemorrhages on the shanks and unfeathered parts of the body and moderate to severe depression. After intravenous (IV) injection, LP infected-birds showed transient mild clinical signs with an intravenous PI (IVPI) of 0.1 (Table 1; Fig. 2 panel b). All chickens injected with LP-Poly and HP died and the calculated IVPI was 3.0 (Table 1; Fig. 2 panel b). Using ELISA, anti-NP antibodies were detected in all surviving chickens inoculated IV and ON with LP or ON with LP-Poly (data not shown).

**Table 1:** Clinical scoring after oculonasal (ON) or intravenous (IV) infection of chickens, turkeys and Muscovy ducks

Virus	Chickens				Turkeys				Muscovy Ducks			
	ON			IV	ON			IV	ON			IV
	Dead/Inoculated^*^	Dead/Contact^*^	PI (MDT; range)	IVPI	Dead/Inoculated	Dead/Contact	PI (MDT; range)	IVPI	Dead/Inoculated	Dead/Contact	PI	IVPI
LP	0/6	0/4	0.0	0.1	0/10	0/5	0.2	0.1	0/10	0/5	0.0	0.0
LP-Poly	5/6	3/4	1.8 (5; 3–9)	3.0	3/10	2/5	1.1 (5; 5)	1.9	0/10	0/5	0.0	0.2
HP	6/6	4/4	2.2 (4.3; 3–5)	3.0	10/10	5/5	2.2 (5; 5)	2.8	0/10	0/5	0.1	0.5

^*^Number of dead birds to the total number of inoculated or contact birds. ON = oculonasal, IV = intravenous, PI = pathogenicity index, IVPI = intravenous pathogenicity index, MDT = mean death time for primarily inoculated birds, “range” refers to the first and last day when birds died. Birds were observed daily and the severity of clinical signs were given scores from 0 (no signs) to 3 (dead). PI and IVPI values range from 0 (avirulent) to 3 (high virulent) and were calculated by dividing the sum of the arithmetic mean values of daily scores of inoculated birds by 10 (the number of observation days) according to the OIE protocol^56^.

Virus excretion was quantified using RT-qPCR of swab samples collected at 4 days post inoculation (dpi). All chickens excreted viruses in oropharyngeal (OP) and cloacal (CL) swabs (Fig. 2 panels c–f). LP virus was detected in the oropharyngeal swabs of inoculated chickens and their cagemates at lower titres than in cloacal swabs (Fig. 2 panels c–f). The insertion of a polybasic HACS significantly (P < 0.05) increased LP excretion in oropharyngeal swabs to a level comparable to HP (Fig. 2 panel c).

**Figure 3:**
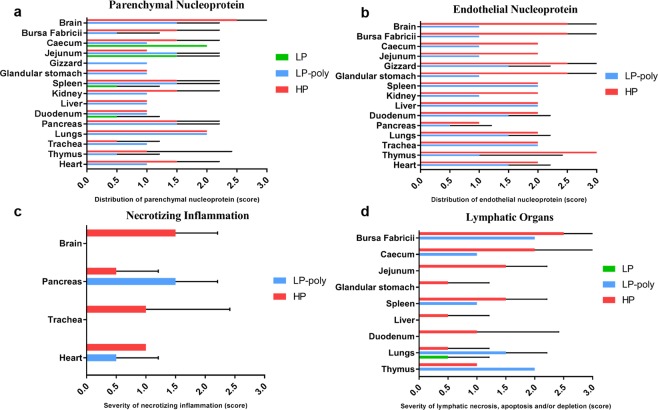
Distribution of influenza A antigen in different organs of inoculated chickens. Distribution of NP antigen in the parenchyma (**a**) and endothelium (**b**) of different organs of two inoculated chickens at 4 days post inoculation was detected by immunohistochemistry. The intensity and distribution of NP signals are scaled 0 (no signal), 1 (focal to oligofocal), 2 (multifocal) or 3 (confluent to diffuse). The severity of lymphatic necrosis, apoptosis and/or depletion (**c**) and necrotizing inflammation (**d**) was assessed by histopathological examination on a scale 0 (negative), 1 (low), 2 (moderate) or 3 (high). Values are shown as median and standard deviation scores of two chickens.

**Figure 4:**
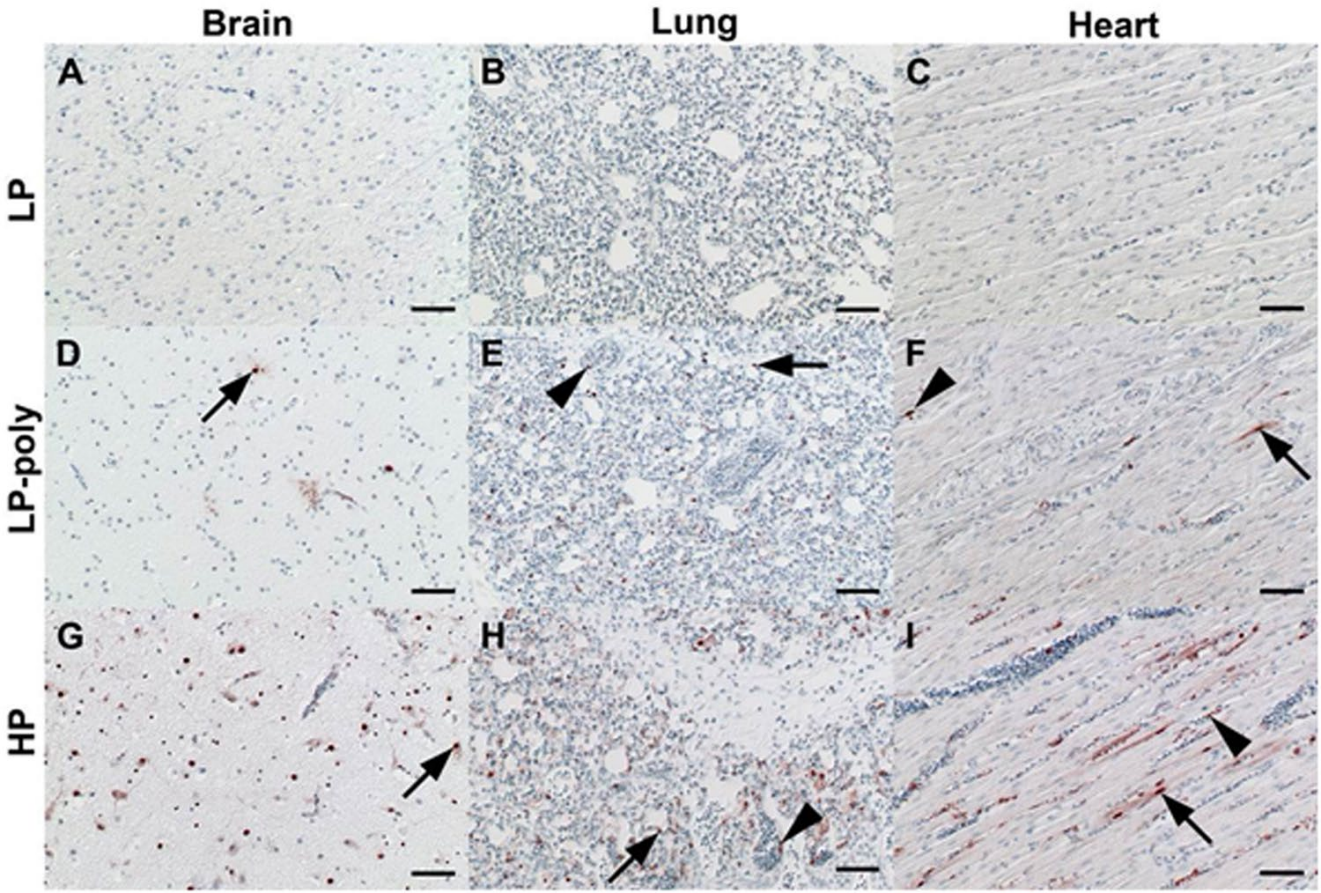
Distribution of influenza A NP antigen in different organs in chickens. Distribution of influenza A NP antigen in different organs in chickens at 4 days post inoculation with LP (**a**–**c**), LP-Poly (**d**–**f**) or HP (**g**–**i**) displaying variable level of organ tropism ranging from a minimum of none to a maximum of coalescing nucleoprotein within brain (**a**,**d**,**g**), lung (**b**,**e**,**h**) and heart (**c**,**f**,**i**). Arrows point to parenchymal cells with influenza A virus nucleoprotein-positive nuclei (neuroglia cells, pneumocytes or cardiomyocytes). Arrowheads point to endothelial cells with influenza A virus nucleoprotein-positive nuclei. Immunohistochemistry; avidin-biotin-peroxidase complex method; polyclonal rabbit anti-influenza A FPV/Rostock/34-virus-nucleoprotein antiserum^61^; 3-amino-9-ethyl-carbazol (red-brown); hematoxylin counterstain (blue); Nomarski contrast; Bars = 50 µm.

On day 4 after ON inoculation, viral NP antigen was detected by immunohistochemistry (IHC) and histology in organs of two chickens per group (Figs 3 and 4). In parenchymal tissues, LP was detected in the intestinal tract (duodenum, jejunum and cecum) and spleen (Fig. 3 panel a). Insertion of the polybasic CS resulted in the distribution of the LP-Poly in all analysed organs at a level comparable to HP. Only LP-Poly was detected in the gizzard. The highest amount of HP and LP-Poly was detected in the brain and lungs (Fig. 3 panel a). LP was not detected in any endothelial tissue, whereas LP-Poly was present in the endothelium of all tissues resembling HP infection (Fig. 3 panel b and Fig. 4). Moreover, histopathological examination of birds inoculated with LP revealed mild inflammation in the lungs only (Fig. 3 panel d). Conversely, HP and LP-Poly induced mild to severe necrosis or necrotic inflammation in the Bursa of Fabricius, thymus, cecum, spleen (Fig. 3 panel d), pancreas and heart (Fig. 3 panel c). Moreover, only HP caused lesions in the jejunum, proventriculus, duodenum, liver, brain and trachea (Fig. 3 panels c and d).

### In turkeys, the LP with polybasic HACS exhibited lower virulence than in chickens and was not detected in the endothelium of any organ

After ON inoculation of turkeys with LP transient mild clinical signs (i.e. ruffled feather and slight depression) were observed in inoculated and contact animals which quickly recovered. None of the turkeys died. All turkeys inoculated ON with HP showed severe depression and central nervous signs (i.e. opisthotonus, torticollis, paralysis) at 4 dpi. They were humanely killed and scored as dead at 5 dpi resulting in a pathogenicity index (PI) of 2.2. All contact turkeys died within 7 days. Interestingly, only 3/10 inoculated and 2/5 contact turkeys died after ON inoculation with LP-Poly. The PI value for this virus was 1.1 which was significantly less than in chickens (P < 0.05) (Fig. 2 panel a). As was observed in chickens, the IVPI of LP and HP were 0.1 and 2.8, respectively, whereas LP-Poly killed only 5 out of 9 birds with an IVPI of 1.9 which was remarkably less than in chickens (Table 1; Fig. 2 panels a and b). All surviving turkeys seroconverted as demonstrated by the detection of anti-NP antibodies by ELISA (data not shown).

Viruses were detected at similar levels in oropharyngeal swabs from inoculated and contact animals (Fig. 2 panels c and d). Like HP inoculated turkeys, LP-Poly inoculated birds excreted higher amounts of viruses via the cloacal route than LP inoculated birds, whereas in-contact turkeys excreted LP-Poly at higher levels than either LP or HP. Compared to chickens, inoculated turkeys oropharyngeal excretion of LP-Poly and HP was about 10- to 100- times decreased, whereas LP and HP were found at about 10 times decreased amounts in the cloacal swabs. LP-Poly and HP were detected at comparable amounts in both cloacal and oropharyngeal swabs, but contact turkeys excreted LP-Poly in cloacal swabs at higher levels than LP and HP (Fig. 2 panels c–f). LP was detected in the cloacal swabs of 4/10 and 2/5 inoculated and contact turkeys, respectively, and in oropharyngeal swabs of all turkeys. All turkeys excreted LP-Poly and HP in cloacal and oropharyngeal swabs.

**Figure 5:**
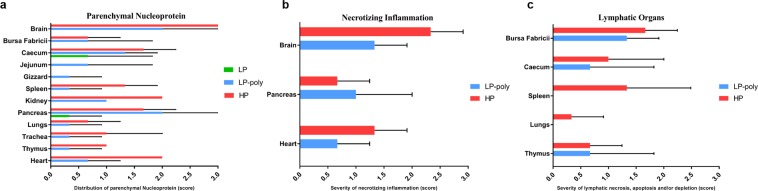
Distribution of influenza A antigen in different organs of inoculated turkeys. Distribution of NP antigen in the parenchyma (**a**) of different organs of three inoculated turkeys at 4 days post inoculation was detected by immunohistochemistry. NP was not detected in the endothelium of any organ of any of the three examined turkeys. The intensity and distribution of NP signals are scaled 0 (no signal), 1 (focal to oligofocal), 2 (multifocal) or 3 (confluent to diffuse). The severity of necrotizing inflammation (**b**) and lymphatic necrosis, apoptosis and/or depletion (**c**) was assessed by histopathological examination on a scale 0 (negative), 1 (low), 2 (moderate) or 3 (high). Values are shown as median and standard deviation scores of three turkeys.

**Figure 6:**
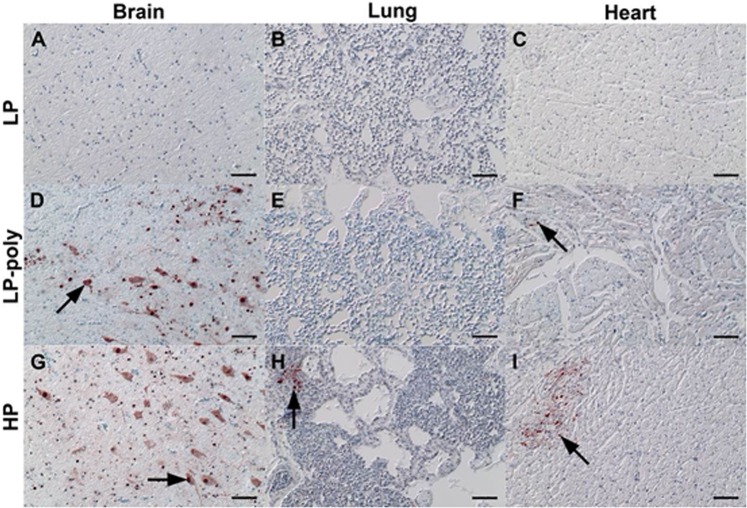
Distribution of influenza A NP antigen in different organs in turkeys. Distribution of influenza A NP antigen in different organs in turkeys at 4 days post inoculation with LP (**a**–**c**), LP-Poly (**d**–**f**) or HP (**g**–**i**) displaying variable level of organ tropism ranging from a minimum of none to a maximum of coalescing nucleoprotein within brain (**a**,**d**,**g**), lung (**b**,**e**,**h**) and heart (**c**,**f**,**i**). Arrows point to parenchymal cells with influenza A virus nucleoprotein-positive nuclei (neuroglia cells, macrophages/pneumocytes or cardiomyocytes). Immunohistochemistry; avidin-biotin-peroxidase complex method; polyclonal rabbit anti-influenza A FPV/Rostock/34-virus-nucleoprotein antiserum 61; 3-amino-9-ethyl-carbazol (red-brown); hematoxylin counterstain (blue); Nomarski contrast; Bars = 50 µm.

Using IHC, a focal to oligofocal distribution of the NP antigen of LP was observed only in the cecal epithelium and parenchyma of pancreas. The distribution of LP-Poly in the parenchyma of different organs was similar to HP, although at slightly lower levels (Figs 5 and 6). The highest amount of LP-Poly was detected in the brain, cecum and pancreas, while HP was concentrated in the brain, cecum, kidneys and heart. Interestingly, like in chickens, only LP-Poly was detected in the gizzard parenchyma in addition to the jejunum (Fig. 5 panel a). Remarkably, and in stark contrast to chickens, none of the viruses were detected in the endothelium of any organ of examined turkeys (Fig. 6). No pathological changes were observed in turkeys inoculated with LP at 4dpi. LP-Poly and HP caused comparable lymphatic necrosis, apoptosis and/or depletion in the bursa, cecum and thymus and comparable mild to severe necrotic encephalitis, pancreatitis and myocarditis (Fig. 5 panels b and c). Nevertheless, only HP caused low to severe necrosis in the spleen and lungs (Fig. 5 panel c).

### Muscovy ducks were more susceptible than Pekin and Mallard ducks and excreted virus at significantly higher levels

Virulence of HP was assessed in Pekin, Mallard and Muscovy ducks after ON and/or IV infection. After ON inoculation of Muscovy ducks with LP, LP-Poly and HP, five sentinel Muscovy ducks were added to assess bird-to-bird transmission. Ducks that were oculonasally inoculated with LP and LP-Poly did not show any clinical signs or mortality. After ON challenge with HP, only Muscovy ducks exhibited a transient mild depression with a PI 0.1, while Pekin and Mallard ducks remained clinically healthy. None of the contact Muscovy ducks showed clinical signs. After IV injection, only Muscovy ducks exhibited mild to moderate depression, reluctance to move and steady gait after infection with HP, and to a lesser extent with LP-Poly with IVPI values of 0.5 and 0.2, respectively (Table 1).

**Figure 7:**
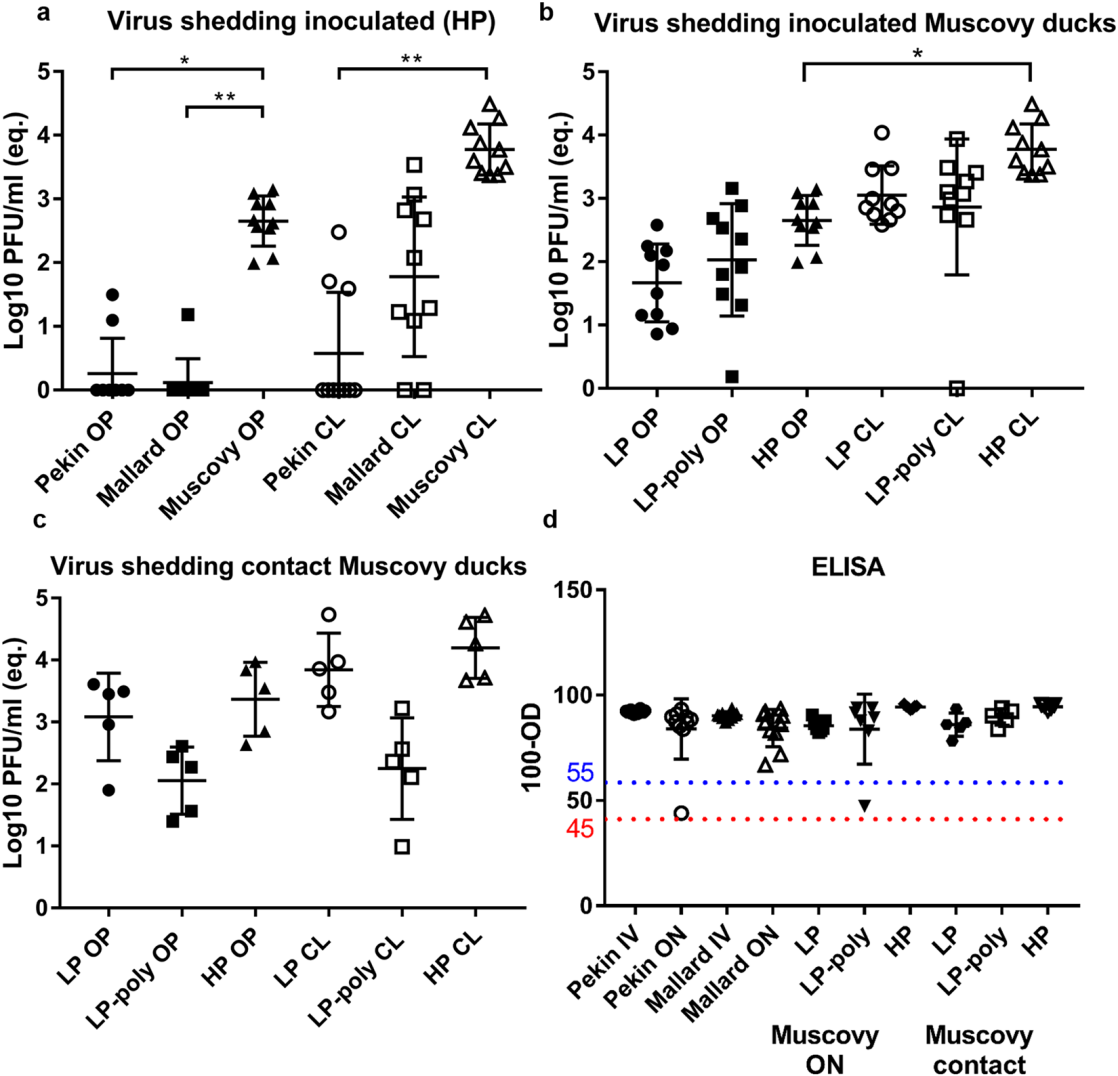
Virus excretion and seroconversion in inoculated and/or contact Pekin, Mallard and Muscovy ducks. Shown are the results of virus excretion at 4 days post inoculation in oropharyngeal (OP) and cloacal (CL) swabs using RT-qPCR expressed by equivalent Log10 PFU/mL in oculonasal (ON) inoculated Pekin, Mallard and Muscovy ducks with HPAIV H7N7. (**a**) Excretion of LP, LP-Poly or HP in inoculated (**b**) and contact (**c**) Muscovy ducks were also determined by RT-qPCR. All values are shown as mean and standard deviation of positive samples. Antibody titres at the end of the experiment in ducks inoculated ON or intravenously (IV) were measured by an NP antibody inhibition ELISA at the end of the experiments. Results are shown as 100- optical density (OD) reading. Lower and upper dashed lines indicate the 45–55% cut-off ratio where samples lower than 45% are negative, from 45 to 55% are questionable and over 55% are positive. (**d**) Statistical significance shown in asterisk indicate P values ≤ 0.05 (*), ≤ 0.01 (**), ≤ 0.001 (***) or ≤ 0.0001 (****).

HP was detected in cloacal swabs at higher levels than in the oropharyngeal swabs in all duck breeds (Fig. 7 Panel a). Pekin ducks excreted the lowest amount of virus and presented the lowest number of shedders. Eight Mallard ducks were positive in cloacal swabs but only one duck was positive in oropharyngeal swabs. Mallard ducks presented higher amounts of virus in cloacal than Pekin ducks. All Muscovy ducks excreted significantly higher amounts of HP oropharynx compared to Mallards and Pekin ducks (Fig. 7 Panel a). Moreover, significantly higher virus titres were found in cloacal swab samples of the Muscovy ducks compared to Pekin ducks. After the ON inoculation with HP, LP, and LP-Poly, viral RNA was detected in oropharyngeal and cloacal swabs from all inoculated Muscovy ducks except one LP-Poly inoculated duck (Fig. 7 Panel b). The average amount in the cloacal swabs was higher than in the oropharyngeal swabs of all viruses (Fig. 7 Panel b). All contact Muscovy ducks were positive in oropharyngeal and cloacal swabs but HP and LP viruses were present in higher amounts in oropharyngeal and cloacal swabs of contact birds compared to LP-Poly (Fig. 7 Panel c). At the termination of the experiment all ON and IV inoculated Pekin and Mallard ducks had anti-NP antibodies as detected by ELISA. Likewise, all Muscovy ducks infected with LP, LP-Poly and HP as well as contact animals seroconverted (Fig. 7 Panel d).

NP detection by IHC was done only for Muscovy ducks. Antigen was not detected in endothelial cells in any Muscovy duck and neither microscopic lesions nor antigen were found in the gizzard or brain. After inoculation with LP, a focal distribution of NP was detected in the epithelium of cecum and trachea (score 1). Histopathological changes were observed in at least one bird including lymphohistiocytic infiltration in the trachea and proventriculus with mild lymphoid depletion in the bursa of Fabricius and thymus. Inoculation of Muscovy ducks with LP-Poly resulted in focal distribution of NP antigens in the parenchyma of thymus, lung, jejunum, cecum and/or bursa of Fabricius of at least one bird. Furthermore, trachea had moderate multifocal degeneration of the epithelium with loss of cilia with lymphocytic infiltration of the lamina propria. In the lungs, hyperplasia of bronchial associated lymphocytes (BALT) and bronchitis with epithelial degeneration and neutrophils infiltration were seen. The pancreas, liver and kidneys contained a mild focal lymphohistiocytic infiltrate and the duodenum had focal lymphocytic aggregation in the gut-associated lymphocytes (GALT). Ducks inoculated with HP had antigen prominently in the thymus and trachea. The trachea exhibited moderate multifocal epithelial degeneration with loss of cilia and lymphocytic infiltration. In the lungs, mild interstitial oedema and congestion with mild lymphatic depletion of BALT was observed. Moreover, mild, acute, oligofocal lymphohistiocytic infiltration in the pancreas and mild lymphoid depletion in the proventriculus were found (data not shown).

## Discussion

In the last few years evidence for direct evolution of HP H7 from LP precursors has been reported^14–16,38^. H7N7 viruses have been very prominent (excluding the current panzootic H5Nx Goose/Guangdong outbreak) during the last decades in Europe causing diseases in poultry and humans^39^. However, little is known about the mechanism underlying the shift of virulence of H7 viruses particularly in turkeys and ducks. In 2015, both HP and LP H7N7 viruses were isolated simultaneously from two neighbouring chicken layer flocks in Germany representing a rare natural isogenic precursor-progeny LPAIV/HPAIV pair. Compared to the LP precursor, the resulting HP had acquired a polybasic HACS but specified, in addition, 10 amino acids alterations namely E123K, I147V, K355R in PB2, F254C in PB1, K185R in PA, I13S in HA, S478F in NP, V439A in NA, V68L in M2 and N92D in NS1 37. In this study, the virulence of the pair of LP and HP viruses, and the impact of the polybasic HACS on virulence and transmission in chickens, turkeys and ducks were investigated.

In previous studies, we have shown that three German LP H7N7 viruses from 2003, 2011 and 2013 required only a specific polybasic HACS to exhibit full virulence equivalent to a genuine HPAIV^12^. Conversely, the insertion of different polybasic HACS motifs in an LP H7N7 isolated in 2001 from a small backyard turkey flock in Germany^12^ and in an Italian LP H7N1 virus of 1999^8^ did not result in full virulence of these viruses. Similarly, several H5N1 and H5N2 viruses with authentic or synthetic polybasic HACS were avirulent in chickens^9,40^. Other mutations in the HA1, HA2, NA or other viral genes were required for expression of high virulence in inoculated and/or sentinel chickens^9,41,42^. We show here that the insertion of the polybasic HACS increased virulence of the LP in chickens to a level comparable with the HP as indicated by IVPI and PI values. While LP was limited to the respiratory and intestinal tracts, insertion of the polybasic HACS resulted in efficient proteolytic activation *in vitro* and systemic distribution resembling the HP infection particularly in the lungs, brains and endothelial cells. These results indicate that the polybasic HACS is the main virulence determinant of this HP H7N7 in chickens, and that other mutations play at most a minimal role in virulence and tropism.

In turkeys, the virulence of LP and HP was comparable to or slightly higher than in chickens. Turkeys are more vulnerable to LP and HP AIV^43,44^ but it is largely unknown whether virulence markers in both “galliform” species are different. Although it is widely accepted that chickens and turkeys are similar in their high susceptibility to AIV, these hosts are not identical. For example, chickens exhibit high susceptibility to Newcastle disease virus, infectious bursal disease virus and infectious laryngeotracheitis virus with severe morbidity and mortality. Nevertheless, these viruses are apathogenic or significantly less virulent in turkeys^45–47^. In the current study, we demonstrate that insertion of a polybasic HACS increased virulence, excretion, tropism and transmission of the LP virus in turkeys. However, this LP-Poly was significantly less virulent than in chickens as demonstrated by lower IVPI and PI values. Intriguingly, only HP-and LP-Poly-inoculated turkeys exhibited neurological signs and no viruses were detected in the endothelial cells of any organ in turkeys suggesting a different pathogenesis of HPAIV in turkeys compared to chickens. This could be the result of virus spread to the brain via the nervous system in turkeys and via the blood stream in chickens. These results are congruent with recent findings of Pantin-Jackwood, *et al*.^43^ who reported neurological signs in turkeys but not in chickens or Mallards after the inoculation with HPAIV H7N8 from the 2016-outbreak in the USA. Therefore, the contribution of host factors to the pathogenesis of H7 viruses in turkeys merits further investigations.

Here, we demonstrated that Mallard and Pekin ducks excreted considerable amounts of HP without showing clinical signs supporting the role of anatid ducks as reservoir for HPAIV^21,28^. It is important to mention that all gene segments of the precursor LP H7N7 were closely related to AIV of wild duck origin indicating a high level of adaptation to ducks^37^ (unpublished data). Interestingly, LP also replicated in and was excreted from Muscovy ducks at the same high level as HP. A higher susceptibility of Muscovy ducks compared to Pekin or Mallard ducks to the 2013 Asian LPAIV H7N9 has been described^48^. Generally, independent of the duck species, excretion through the cloaca was higher than via the oral cavity, which is in accordance with the preferential replication of some AIV in the digestive tract of ducks^49,50^. The IVPI of HP H7N7 in Muscovy ducks was 0.5 indicating moderate virulence. LP-Poly exhibited a slight increase in virulence but was still slightly less virulent than field isolated HPAIV H7N7. This demonstrates that acquisition of the polybasic HACS is the crucial mutation in the transition of LP to HP AIV. However, it also shows that other mutations play a role in determining, in a species-specific manner, high pathogenicity. Contribution of mutations in PA or PB1, in addition to HA, to the high virulence and/or efficient transmission of HPAIV H5N1 in ducks was reported^33–36,51,52^. Resistance of ducks, particularly Mallards, to HPAIV H5N1 was linked to several host immune factors including interferon-induced transmembrane proteins (IFITM) and retinoic acid-inducible gene I (RIG-I) which are absent in chickens^53,54^. Therefore, it is interesting to study the variation of these host factors in different duck species, particularly in Muscovy ducks.

In summary, the recent German HP H7N7 exhibited comparably high virulence in chickens and turkeys, while Pekin and Mallard ducks did not show overt clinical signs. Infected Muscovy ducks developed moderate illness. Tropism of H7N7 viruses to the endothelium was observed in chickens but not in turkeys or ducks. Insertion of a polybasic cleavage site into the HA of the precursor LPAIV H7N7 resulted in increased virulence to different levels in a species-specific manner. In chickens, virulence of LP carrying a polybasic HACS was comparable to the field isolated HPAIV H7N7 supporting the relevance of acquisition of a polybasic cleavage in the transition from LP to HP AIV in chickens. However, in turkeys (and to lesser extent in Muscovy ducks) the virulence increased but significantly less than in chickens suggesting the contribution of other viral or host factors in virulence and pathogenesis of H7N7 in these two species.

## Materials and Methods

### Viruses and cells

LP and HP H7N7 isolated from two commercial layer flocks in Emsland, Germany^37^ were obtained from the National Reference Laboratory for Avian Influenza, Friedrich-Loeffler-Institut (FLI), Greifswald Insel-Riems, Germany. MDCKII used for virus titration and in combination with HEK293T cells for virus rescue were obtained from the Cell Culture Collection in Veterinary Medicine of the FLI. Primary CEK cells were prepared from 18-day-old embryonated chicken eggs as previously done^55^ with few modification. Briefly, embryos were decapitated and the kidneys were obtained using sterile scissors and forceps. After two times incubation with warm trypsin at 37 °C for 20 to 25 minutes, the cells were isolated through a sterile gauze. After centrifugation at 1200 rpm for 5 minutes, the cell pellet was resuspended in minimum essential medium (MEM) containing 10% foetal calf serum (FCS), antibiotics (penicillin and streptomycin) and anti-mycotics (amphotericin).

### Virus propagation

All viruses were propagated in the allantoic sac of SPF ECE purchased from VALO BioMedia GmbH (Osterholz-Scharmbeck, Germany) according to the standard protocol^56^. The allantoic fluid was collected and the hemagglutination activity was measured using 1% chicken erythrocytes^56^. Aliquots of virus stocks were kept at −70 °C until use. Viruses with polybasic HACS were propagated and handled in biosafety level 3 (BSL3) laboratory at the FLI.

SPF ECE were inoculated with the supernatant of transfected cells and candled daily. Dead embryos were kept in the refrigerator for at least one day. Subsequently the allantoic fluids were harvested and checked for bacterial contamination by culturing on Columbia sheep blood agar plates (VWR International, Germany).

### Generation of recombinant viruses

Recombinant viruses were generated by reverse genetics as previously described^57,58^. Briefly, RNA was extracted from LP and HP AIV using the Qiagen RNeasy Kit (Qiagen, Germany) and transcribed to cDNA using the Omniscript Reverse Transcription Kit (Qiagen). All gene segments were amplified, cloned into the plasmid pHWS*ccdB*^57^ and transformed into *E*. *coli* TOP10™ (Invitrogen, USA), XL1-Blue™ or SURE2™ (Stratagene, Netherlands). Plasmids containing LP or HP gene segments were extracted using Plasmid Mini or Maxi kits (Qiagen). Moreover, the insertion of polybasic CS resembling the HP was introduced in the HA gene of LP by QuikChange II XL Site-Directed Mutagenesis Kit (Invitrogen). Primers used for mutagenesis are available upon request. All recombinant viruses were rescued in HEK293T and MDCKII co-culture using Lipofectamine® 2000 and OptiMEM (Fischer Scientific, Germany)^57^. Three viruses were successfully generated: LP, HP and LP containing the polybasic CS (designated LP-Poly). Absence of undesired fortuitous mutations in all gene segments of stock viruses was confirmed by Sanger sequencing and comparison to the wild-type viruses using Geneious software (Biomatters, New Zealand).

### Virus titration

Viruses were titrated by plaque assay. Confluent MDCKII cells in 12-well plates were infected with ten-fold serial dilutions of specified viruses for an hour at 37 °C/5% CO_2_. Cells were overlaid with semisolid Bacto^TM^ Agar (BD, France) containing minimal essential medium (MEM), NaHCO_3_, non-essential amino acids, NA-pyruvate and 4% bovine serum albumin (BSA) (MP Biomedicals, USA). For propagation of LPAIV, 2 μg/mL of N-tosyl-L-phenylalanine chloromethyl ketone (TPCK)-treated trypsin (Sigma, Germany) was added. All plates were incubated for three days at 37 °C. Cells were fixed by 10% formaldehyde containing 0.1% crystal violet. Plaques were counted and viral titres were expressed as plaque forming units per ml (PFU/ml). For measurement of plaque size Nikon NIS-Elements software was used. Up to 100 plaques were measured for each recombinant virus and the results are shown as the mean and standard deviation relative to HP virus.

### Replication kinetics

MDCKII and primary CEK cells were infected at a multiplicity of infection (MOI) of 0.001 for one hour at 37 °C/5% CO_2_. The inoculum was removed and the cells were incubated for two minutes with citric acid buffer pH = 3.0 to inactivate the extracellular virions. Cells were washed twice with PBS, covered by MEM containing 0.2% BSA (Sigma) and incubated at 37 °C/5% CO_2_ for 1, 8, 24, 48 and 72 hours post infection (hpi). The cells and supernatant were harvested and stored at −70 °C. The assay was run in triplicates in two independent rounds for each virus in each type of cells. Virus titres were determined using plaque assay and expressed as mean titres with standard deviation.

### Western blot

The HA cleavage-activation of the three viruses was assessed after infection of MDCKII cells with an MOI of 1 using Western Blot^59^. Briefly, cells were harvested after 24 hpi. After washing with PBS and two centrifugation rounds at 6000 rpm/10 minutes, the cell pellet was suspended in Laemmli buffer (Serva, Germany) and PBS (1:1) and incubated at 95 °C for 10 minutes. Finally the cells were centrifuged at 14000 rpm for 5 minutes. The viral proteins were separated from cell lysate by sodium dodecyl sulphate 10% polyacrylamide gels and then electrotransferred to nitrocellulose membranes using a TransBlot cell (BioRad, USA). The H7 AIV proteins were detected using a polyclonal chicken anti-H7N1 serum at a concentration of 1:500 and peroxidase conjugated rabbit anti-chicken IGY^++^ antibodies (Dianova, Germany) at a concentration of 1:20000 in TBS-T. Immunodetection was achieved by chemiluminescence using Clarity^TM^ Western ECL Substrate (BioRad, USA) and images were captured using a Bio-Rad VersaDoc Imaging System and Quantity One software.

### Animal experiments

Animal experiments were performed after approval by the authorized ethics committee of the State Office of Agriculture, Food Safety, and Fishery in Mecklenburg – Western Pomerania. The commissioner for animal welfare at the FLI representing the Institutional Animal Care and Use Committee (IACUC) also approved the animal experiments, which were performed in accordance with the German Regulations for Animal Welfare.

#### Chickens

SPF ECE from white leghorn chickens were purchased from VALO BioMedia GmbH and incubated for 21 days at the hatchery facilities of the FLI. Six week-old chickens were divided in separate groups with ten animals each. Food and water were added *ad-libitum*. For the ON infection 6 chickens were inoculated with 0.2 ml of a virus dilution in PBS containing 10^5^ PFU/chicken via the oculonasal route. At 1 dpi, four sentinel chickens were added to assess virus transmission. The IVPI was measured by IV injection of ten chickens according to the OIE recommendations^56^. All birds were observed daily for ten days and clinical scoring was done on a scale of 0, 1, 2 and 3^8^. Briefly, healthy birds were given score (0), sick birds showing one clinical sign (e.g. diarrhea, nervous manifestations, respiratory disorders) were given score (1), severely sick birds showed more than one clinical sign were given score (2) and dead birds were given score (3). The pathogenicity index (PI) was calculated by dividing the sum of the arithmetic mean values of daily scores by 10 (the number of observation days). The PI for each virus ranged from 0 (avirulent) to 3 (highly virulent).

#### Turkeys

Commercially available one-day-old white-breasted turkeys were purchased and kept at the FLI animal facilities. Six week-old turkeys were inoculated with the different viruses via ON or IV routes. Ten birds were inoculated ON and 1dpi five sentinel turkeys were added to each group. Moreover, eight week-old turkeys were injected IV to determine the IVPI after OIE regulations and clinically scored as mentioned above.

#### Ducks

One-day old Pekin, Mallard and Muscovy ducks were purchased from a local commercial source. At the FLI, faecal samples were collected from all ducks and examined to exclude, among others, infection with AIV and Salmonella spp. Each experimental room contained a swimming pool filled daily with fresh water. Two to three weeks-old, ten (Pekin and Mallard) or 15 (Muscovy) ducks were allocated to separate groups. At day 0, ten birds were inoculated with 0.2 mL containing 10^5^ PFU via the ON route. For assessing transmissibility of LP, LP-Poly and HP in Muscovy ducks, on day 1 after inoculation of ten Muscovy ducks, five sentinel Muscovy ducks were added. All animals were observed for up to 14 days and clinically scored as mentioned above. To determine the IVPI of indicated viruses, ten 4-to-5-week-old ducks from each breed were injected in the wing vein as previously described^56^.

#### Virus excretion

Oropharyngeal (OP) and cloacal (CL) swabs were collected from ON inoculated birds and their contact peers at 4 dpi and stored at −70 °C until use. The quantity of virus excretion in swab samples was determined using generic real-time reverse-transcription polymerase chain reaction (RT-qPCR)^60^. The RNA was extracted from swab media using NucleoSpin® 8/96 Virus PCR Clean-up Core Kit (Macherey & Nagel GmbH, Germany) in Tecan Freedom Evo System (Tecan, Switzerland). Standard curves using HPAIV H7N7 were run in each RT-qPCR round. The relative amount of excreted virus was quantified by plotting the Ct-values in the standard curves and the results are expressed as the average and standard deviation equivalent log_10_ PFU/ml.

#### Serological examination

Blood was collected at the end of the experiments from all surviving birds after euthanization using isoflurane® (CP-Pharma, Germany). Sera were tested for anti-AIV nucleoprotein (NP) antibodies using enzyme-linked immunosorbent assay (ELISA) by ID screen Influenza A Antibody Competition Multispecies kit (IDvet, France). Plates were read in a Tecan® ELISA reader. The cut-off point according to the manufacture guideline was 55%, samples between 45 and 55% were considered questionable and samples lower than 45% were considered negative.

### Histopathology and immunohistochemistry

To determine the distribution of the different viruses in organs from chickens, turkeys and Muscovy ducks inoculated ON with LP, HP and LP-Poly, samples from trachea, lungs, pancreas, heart, liver, spleen, kidneys, proventriculus, gizzard, duodenum, jejunum, cecum, bursa of Fabricius, thymus and brain from primarily infected chickens (n = 2), turkeys (n = 3) and ducks (n = 3) at 4 dpi were fixed in 10% formalin and embedded in paraffin using standard methods. 5 μm sections were stained by hematoxylin and eosin, and screened for histopathological changes, whereas other sections were subjected to immunohistochemical examination using primary rabbit anti-NP (1:750) antibodies and secondary biotinylated goat anti-rabbit IgG1 (1:200) (Vector, USA)^61^. The level of nucleoprotein antigen was estimated on a 0 to 3 scale: 0 = negative; 1 = focal to oligofocal, 2 = multifocal or 3 = confluent to diffuse and the severity of necrotizing inflammation on a scale of 0 to 3: 0 = no obvious change; 1 = mild, 2 = moderate or 3 = severe as done before^62^.

### Statistics

Statistical differences were analysed using a Kruskal-Wallis with post hoc Tukey tests. Results were considered statistically significant at p value ≤ 0.05. All analysis was done by GraphPad Prism software 7.04 (La Jolla California USA, www.graphpad.com).

## Supplementary information

Cleavage-activation of recombinant H7N7 viruses in this study in presence or absence of trypsin.
