# Supplementary material for: Variable impact of the hemagglutinin polybasic cleavage site on virulence and pathogenesis of avian influenza H7N7 virus in chickens, turkeys and ducks
Source: Sci Rep. 2019 Aug 9;9:11556. doi: 10.1038/s41598-019-47938-3 (PMC6689016; doi:10.1038/s41598-019-47938-3)
Supplement: Supplementary file 1 — Cleavage-activation of recombinant H7N7 viruses in this study in presence or absence of trypsin. [file 41598_2019_47938_MOESM1_ESM.docx]

**Variable impact of the hemagglutinin polybasic cleavage site on virulence and pathogenesis of avian influenza H7N7 virus in chickens, turkeys and ducks**

David Scheibner^1^, Reiner Ulrich^2^, Olanrewaju I. Fatola^2^, Annika Graaf^3^, Marcel Gischke^1^, Ahmed H. Salaheldin^1^, Timm C. Harder^1^, Jutta Veits^1^, Thomas C. Mettenleiter^1^ and Elsayed M. Abdelwhab^1#^

^1^Institute of Molecular Virology and Cell Biology, ^2^Department of Experimental Animal Facilities and Biorisk Management, ^3^Institute of Diagnostic Virology, Friedrich-Loeffler-Institut, Federal Research Institute for Animal Health, Südufer 10, 17493 Greifswald-Insel Riems, Germany

**Supplementary Figure S1:** Cleavage-activation of recombinant H7N7 viruses in this study in presence or absence of trypsin.


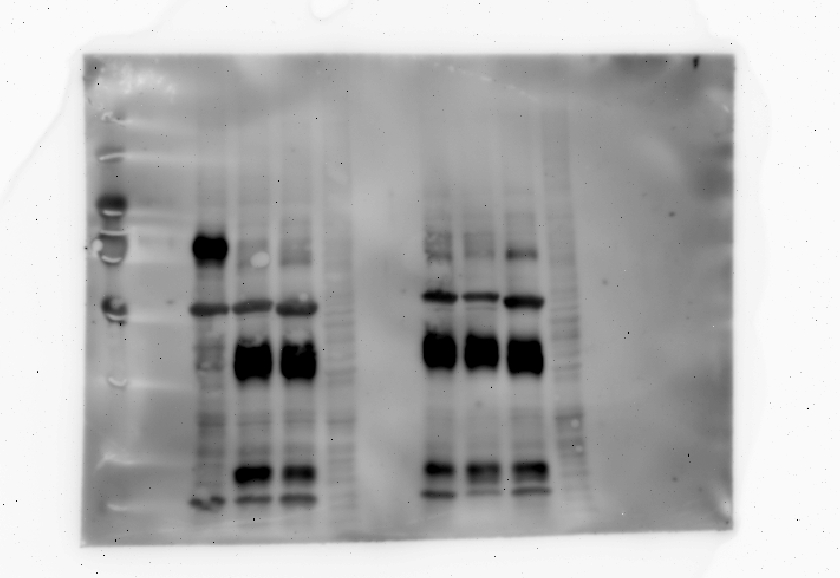


The cleavability of HA0 into HA1 and HA2 subunits was studied using Western Blot after the infection of MDCKII cells at an MOI of 1 PFU per cell of indicated viruses in the presence (+) or absence (-) of trypsin. The viral proteins were detected by polyclonal chicken serum against H7N1 at a ratio 1:500 after separation in a 10% polyacrylamide gel. Shown, from left to right, the protein marker, LP, LP_poly, HP and the mock control (non-infected cells). For full annotation, please refer to Figure 1 panel d.
